# Disparities in not recommending chemotherapy for individuals with newly diagnosed advanced endometrial cancer due to “patient risk factors”

**DOI:** 10.1016/j.gore.2026.102072

**Published:** 2026-03-28

**Authors:** Xuan Li, Allison Dona, Sarah Boyle, Katherine Cooke, Deanna Teoh, Rachel I. Vogel

**Affiliations:** a Department of Obstetrics, Gynecology and Women’s Health, University of Minnesota, Minneapolis, MN, USA; b Masonic Cancer Center, University of Minnesota, Minneapolis, MN, USA

**Keywords:** Health disparities, Endometrial cancer, Chemotherapy, Cancer survivorship

## Abstract

**Objectives:**

We sought to identify patient sociodemographic characteristics associated with not recommending chemotherapy due to “patient risk factors” for individuals diagnosed with advanced-stage endometrial cancer. Methods: We conducted a retrospective analysis of individuals newly diagnosed with stage III or IV endometrial cancer between 2004–2021 utilizing the National Cancer Database (NCDB). The primary outcome was indication that “chemotherapy was not recommended/administered because it was contraindicated due to patient risk factors (i.e., comorbid conditions, advanced age […])” compared to chemotherapy recommended, regardless of administration. A logistic regression model examined associations between patient sociodemographic characteristics (age, race, ethnicity, education, type of insurance, urban/rural residence) and not recommending chemotherapy due to patient risk factors, adjusting for clinical factors including Charlson-Deyo comorbidity index scores, stage, histology, grade, and facility type.

**Results:**

Among the 96,497 individuals included in the analysis, chemotherapy was not recommended for 2,030 (2.1%) due to “patient risk factors.” Increasing age, Black race, living in a zip code with lower rates of high school completion, and having no insurance or government insurance were independently associated with chemotherapy not being recommended after adjusting for clinical variables. Sensitivity analyses excluding subgroups more likely to have clinical contraindications, including those who did not have surgery, had a Charlson-Deyo comorbidity index score > 0, or died within 30 days of surgery found similar results.

**Conclusions:**

Despite National Comprehensive Cancer Network guidelines recommending chemotherapy for frontline treatment of advanced-stage endometrial cancer, disparities in recommendations for chemotherapy were observed by sociodemographic factors including race, age, education, and insurance status.

## Highlights

• NCCN guidelines recommend systemic chemotherapy for frontline treatment of stage III/IV endometrial cancer.
• In the NCDB (2004–2021), for 2.1% of such patients, chemotherapy was not recommended due to “patient risk factors”.
• Increasing age, Black race, less education, and no or government insurance were associated with not recommending chemo.

## 1 Introduction

The National Comprehensive Cancer Network (NCCN) guidelines recommend individuals diagnosed with stage III or IV endometrial cancer receive systemic chemotherapy as part of frontline treatment.(Abu-Rustum et al., 2023) Despite these guidelines, not all patients receive guideline-recommended chemotherapy. Previous studies have highlighted disparities in receiving guideline-concordant therapy for endometrial cancer based on sociodemographic factors such as race, insurance status, and geographic location.(Rauh-Hain et al., 2018, Rodriguez et al., 2021).

Numerous policy, community, institutional, clinical, and personal level factors contribute to differences in receiving chemotherapy. Some of the commonly identified barriers to cancer treatment in high-income countries include insurance and financial constraints, unstable housing, transportation and care location difficulties, barriers to accessing community resources, communication challenges, systemic disintegration, implicit biases, comorbidities, complex psychosocial contexts, and limited social networks.(Bourgeois et al., 2024) Patients may also prefer not to receive chemotherapy because of associated risks or personal beliefs. Cancer care decision-making is a complex process that involves multiple stakeholders, and treatment decisions should be tailored to align with the preferences and goals of the patient.

While socioeconomic factors impact access to and receipt of cancer treatment, the extent to which they impact provider *recommendation* of guideline-concordant chemotherapy is a new area of investigation. Clinical factors, such as comorbidities or poor functional status, can lead to chemotherapy not being recommended because of the morbidity and mortality risk relative to the benefit of the chemotherapy. At the same time, perceived patient characteristics and socioeconomic factors can influence recommendations for guideline-concordant chemotherapy. For patients with early stage invasive breast cancer, a sample of oncologists reported that their decision to recommend adjuvant chemotherapy for patients was impacted by the ability of the patient to follow instructions (59%), social support (38%), and work support/flexibility (36%).(Pini et al., 2012) Differential recommendations have also been documented within gynecologic oncology; among patients with stage II and III ovarian cancer in the National Cancer Database (NCDB) who underwent primary surgery, Matthews et al. found that chemotherapy was not recommended for 1.08% of patients due to “patient risk factors”, and this was more common among Black patients and those older than 70 years.(Matthews et al., 2022) Further, Black patients for whom chemotherapy was not recommended were younger than White patients for whom chemotherapy was not recommended and had a lower overall five-year survival than Black patients who were recommended to receive chemotherapy.(Matthews et al., 2022) This study utilized the NCDB to identify sociodemographic characteristics associated with chemotherapy not being recommended due to “patient risk factors” among patients with newly diagnosed stage III or IV endometrial cancer.

## 2 Methods

We conducted a retrospective cohort study to examine patterns of provider recommendations for chemotherapy among individuals newly diagnosed with stage III or IV endometrial cancer between 2004 and 2021 in the NCDB. This study was deemed exempt by the University of Minnesota Institutional Review Board.

The NCDB is a hospital-based oncology registry capturing approximately 70% of all newly diagnosed cancer cases in the United States.(Winchester et al., 2010) Patients were eligible for this analysis if they had a newly diagnosed stage III or IV invasive endometrial cancer (International Classification of Diseases for oncology, third edition topography C540, C541, C542, C543, C548, C549, C559) between 2004 and 2021. Patients with endometrial stromal sarcomas, histologies associated with non-invasive or non-endometrial cancers, and rare histologies with unique or without standardized treatment recommendations were excluded (Supplemental Table 1).

**Table 1:** NCDB data dictionary for summary of recommendation and receipt of chemotherapy (RX_SUMM_CHEMO).

00	None, chemotherapy was not part of the planned first course of therapy

01	Chemotherapy administered as first course therapy, but the type and number of agents is not documented in patient record
02	Single-agent chemotherapy administered as first course therapy
03	Multiagent chemotherapy administered as first course therapy
82	Chemotherapy was not recommended/administered because it was contraindicated due to patient risk factors (i.e., comorbid conditions, advanced age, progression of tumor prior to administration, etc.)
85	Chemotherapy was not administered because the patient died prior to planned or recommended therapy
86	Chemotherapy was not administered. It was recommended by the patient's physician, but was not administered as part of the first course of therapy. No reason was stated in patient record
87	Chemotherapy was not administered. It was recommended by the patient's physician, but this treatment was refused by the patient, a patient's family member, or the patient's guardian. The refusal was noted in patient record
88	Chemotherapy was recommended, but it is unknown if it was administered
99	It is unknown whether a chemotherapeutic agent(s) was recommended or administered because it is not stated in patient record.

The primary outcome, chemotherapy not recommended due to “patient risk factors”, was defined using the NCDB chemotherapy treatment variable “RX_SUMM_CHEMO”, which indicates recommendation and receipt of chemotherapy (Table 1). Specifically, we compared “Chemotherapy was not recommended/administered because it was contraindicated due to patient risk factors (i.e., comorbid conditions, advanced age, progression of tumor prior to administration, etc.)” to those for whom chemotherapy was recommended, regardless of whether or not chemotherapy was administered (NCDB categorization 82 vs. 1, 2, 3, 85, 86, 87, and 88). Those with a NCDB categorization of 00 (chemotherapy not part of treatment plan [whether chemotherapy was recommended is not specified]) or 99 (whether chemotherapy was recommended /administered is unknown) were excluded from this analysis.

We determined *a priori* the sociodemographic characteristics of interest available within NCDB, including age at diagnosis, race, ethnicity, insurance status, high school noncompletion percentage within the participant’s zip code, and urban/rural residence. The following clinical and tumor characteristics were considered factors that may be associated with both the sociodemographic predictors of interest and the outcome of not recommending chemotherapy: comorbidities (measured as Charlson-Deyo comorbidity index score), cancer stage at diagnosis, histologic subtype (categorized as shown in Supplemental Table 1), tumor grade, year of diagnosis, and facility type.

Demographic and clinical characteristics were summarized using descriptive statistics. A multivariate logistic regression model was conducted to assess the associations between patient sociodemographic variables and not recommending chemotherapy. The sociodemographic variables included age (continuous; truncated at 90 for patients diagnosed at 90 years old or older), race (American Indian/Aleutian/Eskimo, Asian, Black, White, Other, Unknown), ethnicity (Hispanic, non-Hispanic, Unknown), insurance (not insured, private insurance, government insurance, unknown), education (percentage of adults in zip code of residence who did not graduate high school: 21%+, 13–20.9%, 7–12.9%, <7%, not available), and residence (metropolitan, urban, rural, unknown). The model was adjusted for Charlson-Deyo comorbidity index (0, 1, 2, 3+; note patient’s cancer is not directly reflected in the score), tumor stage (III, IV), grade (1, 2, 3, 4, unknown), histologic subtype (carcinosarcoma, clear cell, endometrioid, serous, other as shown in Supplemental Table 1), year of diagnosis (2004–2009, 2010–2015, 2016–2021), and facility type (Community Cancer Program, Comprehensive Community Cancer Program, Academic/Research Program/NCI Comprehensive Cancer Center, or Integrated Network Cancer Program). Adjusted odds ratios (aOR) and 95% confidence intervals (CI) are reported. Analyses were conducted using SAS 9.4 (SAS Institute, Cary, NC).

## 3 Results

A total of 96,497 individuals with newly diagnosed stage III or IV endometrial cancer between 2004 and 2021 were identified in the NCDB and included in this analysis. The median age at diagnosis was 64 (range 18–90+), and most patients were White (77.4%) and lived in metropolitan areas (82.2%; Table 2). The majority had stage III cancer (64.0%), and the most common histology was endometrioid carcinoma (46.5%) followed by serous carcinoma (23.4%). Chemotherapy was not recommended for a total of 2,030 (2.1%) participants due to “patient risk factors”.

**Table 2:** Factors associated with not-recommending chemotherapy due to “patient risk factors” for newly diagnosed advanced-stage endometrial cancer (n = 96,497).

**Characteristic**	**All** **(n = 96,497)**	**Chemotherapy Not Recommended** **(n = 2,030)**	**Chemotherapy** **Recommended (n = 94,467)**	Odds Ratio* (95% CI)	**p-value**
	**Median** **(min, max)**	**Median** **(min, max)**	**Median** **(min, max)**		
Age**(per 5-year increase)	64 (18, 90)	73 (30, 90)	64 (18, 90)	1.42 (1.39, 1.46)	<0.0001
	**n (col %)**	**%**	**%**		
**Race**					<0.0001
American Indian, Aleutian, or Eskimo	363 (0.4)	2.8	97.3	1.74 (0.92, 3.31)	
Asian	3,288 (3.4)	1.5	98.5	1.06 (0.79, 1.42)	
Black	15,729 (16.3)	2.9	97.1	1.46 (1.30, 1.65)	
White	74,646 (77.4)	2.0	98.0	*Ref.*	
Other	1,560 (1.6)	1.5	98.5	1.04 (0.68, 1.58)	
Unknown	911 (0.9)	1.1	98.9	0.56 (0.29, 1.06)	
**Ethnicity**					0.02
Non-Hispanic	86,833 (90.0)	2.2	97.8	*Ref.*	
Hispanic	6,351 (6.7)	1.2	98.8	0.73 (0.57, 0.92)	
Unknown	3,313 (3.4)	2.3	97.7	1.08 (0.85, 1.38)	
**Residence Location**					0.30
Metropolitan	79,297 (82.2)	2.1	97.9	*Ref.*	
Urban	12,178 (12.6)	2.2	97.8	1.01 (0.88, 1.15)	
Rural	1,504 (1.6)	1.9	98.1	0.82 (0.56, 1.19)	
Unknown	3,518 (3.7)	1.5	98.5	0.80 (0.61, 1.05)	
**% adults in zip code who did not graduate high school**					0.04
21.0%+	15,406 (16.0)	2.2	97.8	1.23 (1.05, 1.45)	
13.0–20.9%	21,682 (22.5)	2.2	97.8	1.19 (1.03, 1.37)	
7.0–12.9%	27,778 (28.8)	2.1	97.9	1.14 (1.00, 1.31)	
<7.0%	19,886 (20.6)	1.8	98.2	*Ref.*	
Not available	11,745 (12.2)	2.3	97.7	1.26 (1.07, 1.48)	
**Insurance Status / Type**					<0.0001
Not insured	3,545 (3.7)	1.9	98.1	2.17 (1.66, 2.83)	
Private insurance	38,929 (40.3)	0.9	99.1	*Ref.*	
Government insurance (Medicaid/Medicare/Other)	52,541 (54.5)	3.0	97.0	1.41 (1.24, 1.60)	
Unknown	1,482 (1.5)	1.5	98.5	1.12 (0.72, 1.74)	
**Year of Diagnosis**					<0.0001
2004–2009	18,401 (19.1)	2.5	97.5	*Ref.*	
2010–2015	33,273 (34.5)	2.1	97.9	0.80 (0.71, 0.91)	
2016–2021	44,823 (46.5)	1.9	98.1	0.63 (0.56, 0.71)	
**Charlson-Deyo Comorbidity Index Score**					<0.0001
0	71,481 (74.1)	1.6	98.4	*Ref.*	
1	18,299 (19.0)	2.8	97.2	1.54 (1.38, 1.71)	
2	4,386 (4.6)	3.9	96.1	1.93 (1.64, 2.29)	
3+	2,331 (2.4)	7.8	92.2	3.99 (3.37, 4.72)	
**Stage**					<0.0001
III	61,747 (64.0)	1.7	98.3	*Ref.*	
IV	34,750 (36.0)	2.9	97.1	1.66 (1.51, 1.82)	
**Grade**					<0.0001
1 – Well differentiated	9,227 (9.6)	1.3	98.7	*Ref.*	
2 – Moderately differentiated	16,685 (17.3)	1.5	98.5	1.03 (0.82, 1.28)	
3 – Poorly differentiated	36,308 (37.6)	2.1	97.9	1.21 (0.99, 1.48)	
4 − Undifferentiated	6,634 (6.9)	2.6	97.4	1.62 (1.26, 2.08)	
Unknown/Not specified	27,643 (28.7)	2.6	97.4	1.53 (1.24, 1.88)	
**Histology**					<0.0001
Carcinosarcoma	13,746 (14.3)	3.0	97.0	0.82 (0.72, 0.94)	
Clear cell	3,599 (3.7)	2.5	97.5	0.65 (0.52, 0.82)	
Endometrioid	44,892 (46.5)	2.0	98.0	*Ref.*	
Serous	22,614 (23.4)	1.7	98.3	0.44 (0.39, 0.50)	
Other	11,646 (12.1)	2.0	98.0	0.74 (0.63, 0.86)	
**Facility Type**					<0.0001
Community Cancer Program	3,404 (3.5)	2.2	97.8	1.29 (1.01, 1.65)	
Comp. Community Cancer Program	31,390 (32.5)	2.4	97.6	1.41 (1.27, 1.57)	
Academic/Research Program/ NCI Comp. Cancer Center	39,987 (41.4)	1.7	98.3	*Ref.*	
Integrated Network Cancer Program	19,814 (20.5)	2.6	97.4	1.47 (1.31, 1.66)	
Not available	1,902 (2.0)	0.6	99.4	3.55 (1.90, 6.62)	

* not recommended chemotherapy vs. recommended; adjusted for all variables shown in the table.

** truncated at 90 for individuals > 90 years old at diagnosis.

After adjustment for clinical factors, several sociodemographic factors, including age, race, education, and insurance type, were independently associated with greater odds of chemotherapy not being recommended due to “patient risk factors” (Table 2). The percentage of patients not recommended chemotherapy increased with age, with 0.8% of patients < 50 years old at diagnosis not receiving a recommendation for chemotherapy, 1.3% of those 50–69 years old, 3.6% of those 70–89 years old, and 19.7% of those 90 + years old. After adjusting for known clinical, demographic, and structural factors, older age was associated with increased odds of chemotherapy not being recommended (aOR: 1.42 [95% CI: 1.39, 1.46] per five-year increase in age). Black patients had greater odds of chemotherapy not being recommended compared to White patients (2.9% vs. 2.0%; aOR: 1.46 [95% CI: 1.30, 1.65]). American Indian or Alaska Native individuals had comparably greater frequency and odds of not being recommended chemotherapy than White patients; however, these findings were not precise (2.8% vs. 2.0%, aOR 1.74 [95% CI 0.92, 3.31]). Hispanic patients included in this sample had lower odds of not being recommended chemotherapy compared to non-Hispanic patients (1.2% vs. 2.2%, aOR 0.73 [95 CI 0.57, 0.92]). Additionally, living in an area with lower high school educational attainment (e.g. 21.0% or more of adults in a zip code who did not graduate high school compared to 7% or less, 2.2% vs. 1.8%, aOR: 1.23 [95% CI: 1.05, 1.45]) and having either no insurance (1.9% vs. 0.9%, aOR: 2.17, [95% CI: 1.66, 2.83]) or government-sponsored insurance (Medicaid or Medicare) (3.0% vs. 0.9%, aOR: 1.41 [95% CI: 1.24, 1.60]) compared to private insurance were independently associated with greater odds of chemotherapy not being recommended. While outside the scope of the present research question, several clinical covariates—year of diagnosis, comorbidity index score, stage, grade, histology, and facility type—were also associated with differences in chemotherapy recommendation.

To assess for potential differences in effect between the overall sample and a subsample of those less likely to have an unmeasured clinical contraindication to chemotherapy, we conducted a sensitivity analysis excluding those with Charlson-Deyo comorbidity index score > 0, those who did not have primary surgery, and those who died within 30 days of diagnosis. Not recommending chemotherapy was lower in this sub-population (1.1%); however, the associations between sociodemographic characteristics and not recommending chemotherapy were similar to the overall sample with the exception of education and government insurance, which were not associated with chemotherapy recommendation in this subgroup (Supplemental Table 2). We additionally excluded patients 80 years or older and the conclusions remained the same (data not shown).

## 4 Discussion

This study identified significant sociodemographic disparities in chemotherapy recommendations for advanced-stage endometrial cancer, despite NCCN guidelines. Older age, Black race, lower community education levels, and non-private insurance were independently linked to higher odds of chemotherapy being withheld due to “patient risk factors.” Given that disparities persisted after adjustment for clinical factors that impact the level of risk and recommendation of chemotherapy (e.g. comorbidity score, facility type), there is a lack of clinical-based factors to account for these disparities.

Our results add to the broader understanding of disparities in cancer treatment. Similar work using NCDB data in stage II-III ovarian cancer also found that Black and older adults as well as those who had Medicare or other non-income-based government insurance were less likely to be prescribed guideline-concordant chemotherapy because of “patient risk factors”.(Matthews et al., 2022) Education and ethnicity were, however, not included in their core model.(Matthews et al., 2022) Our findings suggest that, in addition to known structural barriers to accessing recommended treatment, sociodemographic disparities in chemotherapy recommendations also extend to endometrial cancer and may represent a broader trend in chemotherapy decision-making.

Because clinical factors do not fully explain these disparities, the influence of sociodemographic factors on provider recommendations for guideline-concordant care must be considered. Healthcare discrimination is commonly reported by patients across the United States, including those with gynecologic cancer.(Dona et al., 2025, Nong et al., 2020, Arditi et al., 2023) Conscious and implicit biases related to age, socioeconomic status, race, and/or perceived treatment tolerance contribute to disparities in care.(Hall et al., 2015) This may contribute to differential estimations of risk, which could ultimately lead to the differences in recommending guideline-concordant chemotherapy observed in our study.

Addressing these disparities will require a comprehensive approach that integrates patient, provider, institutional, and systemic factors to ensure that all patients with advanced-stage endometrial cancer receive appropriate and equitable oncologic care. From a systems perspective, evidence supports that improving access to high-quality, evidence-based care can diminish disparities and potentially close the racial disparity gap seen in survival for gynecologic cancers.(Rauh-Hain et al., 2018) Differences in covariates such as facility type further emphasize the potential impact of policy, resource access, and other systemic factors on recommendation of chemotherapy. Institutional strategies may involve strengthening referral pathways to gynecologic oncologists to ensure timely access and enforcing guideline concordant therapies. From a provider perspective, studies have shown that implicit bias against Black, Hispanic, and dark-skinned individuals is prevalent among health care providers regardless of specialty and level of experience.(Hall et al., 2015) Therefore, implementing strategies to combat implicit bias such as standardized decision-support tools, multidisciplinary tumor boards, and implicit bias training for providers could help mitigate disparities in chemotherapy recommendations, although more research on interventions to reduce provider bias is needed.(Hall et al., 2015, Kang et al., 2014, Zeidan et al., 2019, Perdomo et al., 2019) At the patient level, this includes addressing socioeconomic barriers and ensuring culturally and linguistically appropriate communication to support informed decision-making.

Despite the large national sample, this study has several limitations. First, the NCDB lacks performance status data, a common clinical driver for withholding chemotherapy. Second, the database excludes some hospitals, potentially introducing selection bias; specifically, Hispanic patients are significantly underrepresented in the NCDB (capture rates of 40.8% in 2004–2006 and 54.8% in 2017–2019)(Satpathy et al., 2023), likely due to structural barriers and limited access to participating facilities. Third, the “patient risk factors” coding is subjective and dependent on medical record documentation. Finally, unmeasured confounding and mediation remain possible, as variables like insurance type lack the nuance to account for varying coverage levels and not all comorbidities are accounted for by the Charlson-Deyo Comorbidity Index Score.

## 5 Conclusion

Despite guideline recommendations, National Cancer Database (NCDB) analysis reveals significant disparities in clinician chemotherapy recommendation for advanced-stage endometrial cancer. Even after adjusting for clinical factors, Black patients, older individuals, those from lower-education areas, and those underinsured or government-insured were less likely to receive recommendations for chemotherapy. These persistent gaps suggest that implicit bias and subjective perceived risk may contribute to treatment inequity in endometrial cancer care.

## CRediT authorship contribution statement

**Xuan Li:** Writing – review & editing, Writing – original draft, Investigation. **Allison Dona:** Writing – review & editing, Writing – original draft, Methodology, Investigation. **Sarah Boyle:** Writing – review & editing, Investigation. **Katherine Cooke:** Writing – review & editing, Investigation. **Deanna Teoh:** Writing – review & editing, Methodology, Investigation, Conceptualization. **Rachel I. Vogel:** Writing – review & editing, Writing – original draft, Supervision, Methodology, Investigation, Formal analysis, Data curation, Conceptualization.

## References

[b0005] Abu-Rustum N, Yashar C, Arend R, et al. Uterine Neoplasms, Version 1.2023, NCCN Clinical Practice Guidelines in Oncology. *Journal of the National Comprehensive Cancer Network : JNCCN*. Feb 2023;21(2):181-209. doi:10.6004/jnccn.2023.0006. 10.6004/jnccn.2023.0006 36791750

[b0010] Rauh-Hain J.A., Melamed A., Schaps D. (2018). Racial and ethnic disparities over time in the treatment and mortality of women with gynecological malignancies. Gynecol. Oncol..

[b0015] Rodriguez V.E., LeBron A.M.W., Chang J., Bristow R.E. (2021). Racial-Ethnic and Socioeconomic Disparities in Guideline-Adherent Treatment for Endometrial Cancer. Obstet. Gynecol..

[b0020] Bourgeois A, Horrill T, Mollison A, Stringer E, Lambert LK, Stajduhar K. Barriers to cancer treatment for people experiencing socioeconomic disadvantage in high-income countries: a scoping review. *BMC Health Serv Res*. May 28 2024;24(1):670. doi:10.1186/s12913-024-11129-2. 10.1186/s12913-024-11129-2 PMC11134650 38807237

[b0025] Pini T.M., Hawley S.T., Li Y., Katz S.J., Griggs J.J. (Jul 2012). The influence of non-clinical patient factors on medical oncologists' decisions to recommend breast cancer adjuvant chemotherapy. Breast Cancer Res. Treat..

[b0030] Matthews B.J., Qureshi M.M., Fiascone S.J. (2022). Racial disparities in non-recommendation of adjuvant chemotherapy in stage II-III ovarian cancer. Gynecol. Oncol..

[b0035] Winchester D.P., Stewart A.K., Phillips J.L., Ward E.E. (2010). The national cancer data base: past, present, and future. Ann. Surg. Oncol..

[b0040] Dona A.C., Jewett P.I., Davidson S., Teoh D., Vogel R.I. (2025). Experience of healthcare discrimination reported by individuals with a history of gynecologic cancer in the all of Us research program. *Gynecologic*. Oncol. Rep..

[b0045] Nong P., Raj M., Creary M., Kardia S.L.R., Platt J.E. (2020). Patient-Reported Experiences of Discrimination in the US Health Care System. JAMA Netw. Open.

[b0050] Arditi C, Eicher M, Junod J, Peytremann-Bridevaux I. Socio-demographic and health-related determinants of patients' overall rating and experiences of cancer care. *BMC Cancer*. Sep 29 2023;23(1):918. doi:10.1186/s12885-023-11445-6. 10.1186/s12885-023-11445-6 PMC10540394 37773108

[b0055] Hall W.J., Chapman M.V., Lee K.M. (2015). Implicit Racial/Ethnic Bias among Health Care professionals and its Influence on Health Care Outcomes: a Systematic Review. Am. J. Public Health.

[b0060] Kang Y., Gray J.R., Dovidio J.F. (2014). The nondiscriminating heart: lovingkindness meditation training decreases implicit intergroup bias. J. Exp. Psychol. Gen..

[b0065] Zeidan AJ, Khatri UG, Aysola J, et al. Implicit Bias Education and Emergency Medicine Training: Step One? Awareness. *AEM Educ Train*. Jan 2019;3(1):81-85. doi:10.1002/aet2.10124. 10.1002/aet2.10124 PMC6339553 30680351

[b0070] Perdomo J., Tolliver D., Hsu H. (2019). Health Equity Rounds: an Interdisciplinary Case Conference to Address Implicit Bias and Structural Racism for faculty and Trainees. MedEdPORTAL. Nov 22.

[b0075] Satpathy Y., Nam P., Moldovan M. (2023). Comparison of Capture rates of the National Cancer Database across Race and Ethnicity. JAMA Netw. Open.

