# Supplementary material for: Disparities in not recommending chemotherapy for individuals with newly diagnosed advanced endometrial cancer due to “patient risk factors”
Source: Gynecol Oncol Rep. 2026 Mar 28;64:102072. doi: 10.1016/j.gore.2026.102072 (PMC13083775; doi:10.1016/j.gore.2026.102072)
Supplement: Supplementary Data 1 [file mmc1.docx]

**Supplemental Table 1. Categorizations and exclusions based on histology.**

| **Histology Code and Description** | **Category** | **Excluded from Analysis** |
| --- | --- | --- |
| 8000-Neoplasm, malignant | Unknown/Not Specified | Yes |
| 8001-Tumor cells, malignant | Unknown/Not Specified | Yes |
| 8004-Malignant tumor, spindle cell type | Sarcoma | Yes |
| 8005-Malignant tumor, clear cell type | Clear cell | No |
| 8010-Carcinoma, NOS | Unknown/Not Specified | Yes |
| 8012-Large cell carcinoma, NOS | Other | Yes |
| 8013-Large cell neuroendocrine carcinoma | Other | Yes |
| 8015-Glassy cell carcinoma | Other | Yes |
| 8020-Carcinoma, undifferentiated, NOS | Other | No |
| 8021-Carcinoma, anaplastic, NOS | Other | No |
| 8022-Pleomorphic carcinoma | Other | Yes |
| 8031-Giant cell carcinoma | Other | Yes |
| 8032-Spindle cell carcinoma, NOS | Other | Yes |
| 8033-Pseudosarcomatous carcinoma | Carcinosarcoma | No |
| 8035-Carcinoma with osteoclast-like giant cells | Other | Yes |
| 8041-Small cell carcinoma, NOS | Other | Yes |
| 8044-Small cell carcinoma, intermediate cell | Other | Yes |
| 8045-Combined small cell carcinoma | Other | Yes |
| 8046-Non-small cell carcinoma | Other | Yes |
| 8050-Papillary carcinoma, NOS | Serous | No |
| 8051-Verrucous carcinoma, NOS | Other | Yes |
| 8052-Papillary squamous cell carcinoma | Other | No |
| 8070-Squamous cell carcinoma, NOS | Other | Yes |
| 8071-Squamous cell carcinoma, keratinizing, NOS | Other | Yes |
| 8072-Squamous cell carcinoma, large cell, nonkeratinizing, NOS | Other | Yes |
| 8073-Squamous cell carcinoma, small cell, nonkeratinizing | Other | Yes |
| 8074-Squamous cell carcinoma, spindle cell | Other | Yes |
| 8075-Squamous cell carcinoma, adenoid | Other | Yes |
| 8082-Lymphoepithelial carcinoma | Other | Yes |
| 8083-Basaloid squamous cell carcinoma | Other | Yes |
| 8084-Squamous cell carcinoma, clear cell type | Other | Yes |
| 8085-Squamous cell carcinoma, HPV positive | Other | Yes |
| 8120-Transitional cell carcinoma, NOS | Other | Yes |
| 8122-Urothelial carcinoma, sarcomatoid | Other | Yes |
| 8130-Papillary urothelial carcinoma | Other | Yes |
| 8140-Adenocarcinoma, NOS | Unknown/Not Specified | Yes |
| 8141-Scirrhous adenocarcinoma | Other | Yes |
| 8144-Adenocarcinoma, intestinal type | Other | No |
| 8190-Trabecular adenocarcinoma | Other | Yes |
| 8200-Adenoid cystic carcinoma | Other | Yes |
| 8201-Cribriform carcinoma, NOS | Other | No |
| 8210-Adenocarcinoma in adenomatous polyp | Other | No |
| 8230-Solid carcinoma, NOS | Unknown/Not Specified | Yes |
| 8240-Neuroendocrine tumor, NOS | Other | Yes |
| 8244-Mixed adenoneuroendocrine carcinoma | Other | Yes |
| 8246-Neuroendocrine carcinoma, NOS | Other | Yes |
| 8249-Neuroendocrine tumor, grade 2 | Other | Yes |
| 8255-Adenocarcinoma with mixed subtypes | Other | No |
| 8260-Papillary adenocarcinoma, NOS | Serous | No |
| 8261-Adenocarcinoma in villous adenoma | Other | No |
| 8262-Villous adenocarcinoma | Other | No |
| 8263-Adenocarcinoma in tubulovillous adenoma | Other | No |
| 8290-Oxyphilic adenocarcinoma | Other | No |
| 8310-Clear cell adenocarcinoma, NOS | Clear cell | No |
| 8312-Renal cell carcinoma, NOS | Other | Yes |
| 8313-Clear cell adenocarcinofibroma | Clear cell | No |
| 8320-Granular cell carcinoma | Other | Yes |
| 8323-Mixed cell adenocarcinoma | Other | No |
| 8380-Endometrioid adenocarcinoma, NOS | Endometrioid | No |
| 8381-Endometrioid adenofibroma, malignant | Endometrioid | No |
| 8382-Endometrioid adenocarcinoma, secretory variant | Endometrioid | No |
| 8383-Endometrioid adenocarcinoma, ciliated cell variant | Endometrioid | No |
| 8384-Adenocarcinoma, endocervical type, NOS | Other | Yes |
| 8410-Sebaceous carcinoma | Other | Yes |
| 8440-Cystadenocarcinoma, NOS | Endometrioid | No |
| 8441-Serous carcinoma, NOS | Serous | No |
| 8450-Papillary cystadenocarcinoma, NOS | Serous | No |
| 8460-Low grade serous carcinoma | Serous | No |
| 8461-High grade serous carcinoma | Serous | No |
| 8463-Serous surface papillary tumor | Other | Yes |
| 8471-Mucinous cystadenocarcinoma | Other | No |
| 8480-Mucinous adenocarcinoma | Other | No |
| 8481-Mucin-producing adenocarcinoma | Other | No |
| 8482-Mucinous carcinoma, gastric type | Other | No |
| 8490-Signet ring cell carcinoma | Other | Yes |
| 8500-Infiltrating duct carcinoma, NOS | Other | Yes |
| 8510-Medullary carcinoma, NOS | Other | Yes |
| 8560-Adenosquamous carcinoma | Other | No |
| 8562-Epithelial-myoepithelial carcinoma | Other | Yes |
| 8570-Adenocarcinoma with squamous metaplasia | Endometrioid | No |
| 8572-Adenocarcinoma with spindle cell metaplasia | Endometrioid | No |
| 8574-Adenocarcinoma with neuroendocrine differentiation | Endometrioid | No |
| 8575-Metaplastic carcinoma, NOS | Other | No |
| 8590-Ovarian stromal tumor | Other | Yes |
| 8640-Sertoli cell carcinoma | Other | Yes |
| 8714-Perivascular epithelioid tumor, malignant | Sarcoma | Yes |
| 8800-Sarcoma, NOS | Sarcoma | Yes |
| 8802-Giant cell sarcoma | Sarcoma | Yes |
| 8803-Small cell sarcoma | Sarcoma | Yes |
| 8805-Undifferentiated sarcoma | Sarcoma | Yes |
| 8890-Leiomyosarcoma, NOS | Sarcoma | Yes |
| 8891-Epithelioid leiomyosarcoma | Sarcoma | Yes |
| 8895-Myosarcoma | Sarcoma | Yes |
| 8896-Myxoid leiomyosarcoma | Sarcoma | Yes |
| 8900-Rhabdomyosarcoma, NOS | Sarcoma | Yes |
| 8901-Pleomorphic rhabdomyosarcoma, adult type | Sarcoma | Yes |
| 8902-Mixed type rhabdomyosarcoma | Sarcoma | Yes |
| 8910-Embryonal rhabdomyosarcoma, NOS | Sarcoma | Yes |
| 8912-Spindle cell rhabdomyosarcoma | Sarcoma | Yes |
| 8920-Alveolar rhabdomyosarcoma | Sarcoma | Yes |
| 8930-Endometrial stromal sarcoma, NOS | Other | Yes |
| 8931-Endometrial stromal sarcoma, low grade | Other | Yes |
| 8933-Adenosarcoma | Sarcoma | Yes |
| 8935-Stromal sarcoma, NOS | Other | Yes |
| 8940-Mixed tumor, malignant, NOS | Carcinosarcoma | No |
| 8950-Mullerian mixed tumor | Carcinosarcoma | No |
| 8951-Mesodermal mixed tumor | Carcinosarcoma | No |
| 8980-Carcinosarcoma, NOS | Carcinosarcoma | No |
| 8981-Carcinosarcoma, embryonal | Carcinosarcoma | No |
| 9110-Mesonephroma, malignant | Other | Yes |
| 9111-Mesonephric-like adenocarcinoma | Other | Yes |

**Supplemental Table 2. Factors associated with not-recommending chemotherapy due to “patient risk factors” for newly diagnosed advanced-stage endometrial cancer, excluding patients with Charleson comorbidity score >0, did not have primary site surgery, or died within 30 days of diagnosis (n=63,182).**

| **Characteristic** | **All**  **(n=63,182)** | **Chemotherapy Not Recommended**  **(n=693)** | **Chemotherapy Recommended (n=62,489)** | **Odds Ratio***  **(95% CI)** | **p-value** |
| --- | --- | --- | --- | --- | --- |
|  | **Median**  **(min, max)** | **Median**  **(min, max)** | **Median**  **(min, max)** |  | <0.0001 |
| **Age** (per 5-year increase)** | 64 (18, 90) | 74 (30, 90) | 64 (18, 90) | 1.56 (1.49, 1.64) |  |
|  | **n (%)** | **%** | **%** |  |  |
| **Race** |  |  |  |  | 0.0002 |
| Asian | 2,297 (3.6) | 0.8 | 99.2 | 1.18 (0.74, 1.88) |  |
| Black | 8,851 (14.0) | 1.4 | 98.6 | 1.61 (1.30, 2.01) |  |
| White | 50,243 (79.5) | 0.7 | 99.3 | *Ref.* |  |
| Other******* | 1,791 (2.8) | 1.1 | 98.9 | 0.87 (0.48, 1.57) |  |
| **Ethnicity** |  |  |  |  | 0.44 |
| Non-Hispanic | 56,914 (90.1) | 1.1 | 98.9 | *Ref.* |  |
| Hispanic | 4,040 (6.4) | 0.6 | 99.4 | 0.76 (0.50, 1.16) |  |
| Unknown | 2,228 (3.5) | 1.4 | 98.6 | 1.00 (0.69, 1.45) |  |
| **Residence Location** |  |  |  |  | 0.18 |
| Metropolitan | 51,865 (82.1) | 1.1 | 98.9 | *Ref.* |  |
| Urban | 8,000 (12.7) | 1.3 | 98.7 | 1.20 (0.97, 1.49) |  |
| Rural | 958 (1.5) | 1.2 | 98.9 | 0.93 (0.51, 1.71) |  |
| Unknown | 2,359 (3.7) | 0.7 | 99.3 | 0.72 (0.44, 1.18) |  |
| **% adults in zip code who did not graduate high school** |  |  |  |  | 0.56 |
| 21.0%+ | 9,466 (15.0) | 1.2 | 98.8 | 1.19 (0.91, 1.55) |  |
| 13.0-20.9% | 13,724 (21.7) | 1.1 | 98.9 | 1.02 (0.80, 1.29) |  |
| 7.0-12.9% | 18,534 (29.3) | 1.1 | 98.9 | 1.03 (0.83, 1.27) |  |
| <7.0% | 14,111 (22.3) | 1.1 | 99.0 | *Ref.* |  |
| Not available | 7,347 (11.6) | 1.1 | 98.9 | 1.18 (0.89, 1.55) |  |
| **Insurance Status / Type** |  |  |  |  | 0.04 |
| Not insured | 2,258 (3.6) | 0.9 | 99.1 | 1.86 (1.17, 2.95) |  |
| Private insurance | 27,983 (44.3) | 0.5 | 99.5 | *Ref.* |  |
| Government insurance (Medicaid/Medicare/Other) | 31,897 (50.5) | 1.6 | 98.4 | 1.17 (0.95, 1.45) |  |
| Unknown | 1,044 (1.7) | 0.7 | 99.3 | 0.80 (0.37, 1.73) |  |
| **Year of Diagnosis** |  |  |  |  | <0.0001 |
| 2004-2009 | 12,878 (20.4) | 1.7 | 98.4 | *Ref.* |  |
| 2010-2015 | 21,901 (34.7) | 1.2 | 98.8 | 0.71 (0.59, 0.85) |  |
| 2016-2021 | 28,403 (45.0) | 0.8 | 99.2 | 0.44 (0.36, 0.53) |  |
| **Stage** |  |  |  |  | 0.24 |
| III | 44,484 (70.4) | 1.1 | 98.9 | *Ref.* |  |
| IV | 18,698 (29.6) | 1.1 | 98.9 | 1.11 (0.93, 1.31) |  |
| **Grade** |  |  |  |  | 0.09 |
| 1 – Well differentiated | 6,602 (10.5) | 0.8 | 99.2 | *Ref.* |  |
| 2 – Moderately differentiated | 11,938 (18.9) | 1.0 | 99.0 | 1.13 (0.81, 1.57) |  |
| 3 – Poorly differentiated | 25,087 (39.7) | 1.3 | 98.7 | 1.27 (0.93, 1.74) |  |
| 4 - Undifferentiated | 4,357 (6.9) | 1.5 | 98.6 | 1.47 (0.99, 2.19) |  |
| Unknown/Not specified | 15,198 (24.1) | 0.9 | 99.1 | 1.03 (0.73, 1.46) |  |
| **Histology** |  |  |  |  | <0.0001 |
| Carcinosarcoma | 8,521 (13.5) | 1.6 | 98.4 | 0.93 (0.74, 1.16) |  |
| Clear cell | 2,224 (3.5) | 1.1 | 98.9 | 0.55 (0.36, 0.85) |  |
| Endometrioid | 30,294 (48.0) | 1.2 | 98.9 | *Ref.* |  |
| Serous | 14,490 (22.9) | 0.8 | 99.2 | 0.40 (0.32, 0.51) |  |
| Other | 7,653 (12.1) | 0.9 | 99.1 | 0.61 (0.47, 0.80) |  |
| **Facility Type** |  |  |  |  | <0.0001 |
| Community Cancer Program | 2,261 (3.6) | 1.2 | 98.8 | 1.34 (0.89, 2.02) |  |
| Comp. Community Cancer Program | 20,771 (32.9) | 1.2 | 98.8 | 1.39 (1.16, 1.67) |  |
| Academic/Research Program/  NCI Comp. Cancer Center | 25,941 (41.1) | 0.8 | 99.2 | *Ref.* |  |
| Integrated Network Cancer Program | 12,832 (20.3) | 1.4 | 98.6 | 1.65 (1.35, 2.01) |  |
| Not available | 1,377 (2.2) | 0.4 | 99.6 | 6.84 (2.68, 17.48) |  |

*truncated at 90 for individuals >90 years old at diagnosis

**not recommended chemotherapy vs. recommended; adjusted for all variables shown in the table

***due to small sample sizes, American Indian, Other and Unknown race combined
